# Transcriptomic Analysis of Stem Cells Treated with Moringin or Cannabidiol: Analogies and Differences in Inflammation Pathways

**DOI:** 10.3390/ijms20236039

**Published:** 2019-11-30

**Authors:** Luigi Chiricosta, Serena Silvestro, Jacopo Pizzicannella, Francesca Diomede, Placido Bramanti, Oriana Trubiani, Emanuela Mazzon

**Affiliations:** 1 Istituto di Ricovero e Cura a Carattere Scientifico Centro Neurolesi “Bonino-Pulejo”, 98124 Messina, Italy; luigi.chiricosta@irccsme.it (L.C.); serena.silvestro@irccsme.it (S.S.); placido.bramanti@irccsme.it (P.B.); 2 Azienda Sanitaria Locale 02 Lanciano-Vasto-Chieti, “Ss. Annunziata” Hospital, 66100 Chieti, Italy; 3 Dipartimento di Scienze Mediche, Orali e Biotecnologiche, Università “G. d’Annunzio” Chieti-Pescara, 66100 Chieti, Italy; francesca.diomede@unich.it (F.D.); trubiani@unich.it (O.T.)

**Keywords:** transcriptome analysis, inflammation, Moringin, Cannabidiol, human gingival mesenchymal stem cells

## Abstract

Inflammation is a common feature of many neurodegenerative diseases. The treatment of stem cells as a therapeutic approach to repair damage in the central nervous system represents a valid alternative. In this study, using Next-Generation Sequencing (NGS) technology, we analyzed the transcriptomic profile of human Gingival Mesenchymal Stem Cells (hGMSCs) treated with Moringin [4-(α-l-ramanosyloxy)-benzyl isothiocyanate] (hGMSCs-MOR) or with Cannabidiol (hGMSCs-CBD) at dose of 0.5 or 5 µM, respectively. Moreover, we compared their transcriptomic profiles in order to evaluate analogies and differences in pro- and anti-inflammatory pathways. The hGMSCs-MOR selectively downregulate TNF-α signaling from the beginning, reducing the expression of TNF-α receptor while hGMSCs-CBD limit its activity after the process started. The treatment with CBD downregulates the pro-inflammatory pathway mediated by the IL-1 family, including its receptor while MOR is less efficient. Furthermore, both the treatments are efficient in the IL-6 signaling. In particular, CBD reduces the effect of the pro-inflammatory JAK/STAT pathway while MOR enhances the pro-survival PI3K/AKT/mTOR. In addition, both hGMSCs-MOR and hGMSCs-CBD improve the anti-inflammatory activity enhancing the TGF-β pathway.

## 1. Introduction

Many neurodegenerative diseases such as Alzheimer’s Disease (AD), Parkinson’s disease (PD), Multiple Sclerosis (MS) and Amyotrophic Lateral Sclerosis (ALS) are characterized by neuro-inflammation that leads to cell death [1]. The regenerative potential of stem cells can be used as a therapeutic strategy to repair damaged nervous tissue [2,3]. The human Gingival Mesenchymal Stem Cells (hGMSCs) show immunomodulatory effect and differentiation capacity [4]. Particularly, hGMSCs originate from the neural crest; thus, they can differentiate into neuronal cells [5]. Moreover, hGMSCs are easy to harvest and exhibit anti-inflammatory and antiapoptotic effects [6,7]. Specifically, inflammatory activities are mediated by the release of extracellular vesicles containing anti-inflammatory cytokines such as Transforming Growth Factor beta (TGF-β) and Interleukin 10 (IL-10).

Our experiment is aimed to evaluate the transcriptomic profile of hGMSCs pre-treatment with two phytochemical compounds, Moringin [4-(α-l-ramanosyloxy)-benzyl isothiocyanate] (MOR) or Cannabidiol (CBD). Therefore, we studied if MOR or CBD can counteract the neuro-inflammation that characterize the pathogenesis of the neurodegenerative diseases exerting their effects in the same or in different pathways.

MOR, extracted from *Moringa oleifera* (fam. Moringaceae), is an isothiocyanate derived from glucomoringin. MOR is bioactivated by myrosinase enzyme, an endogenous β-thioglucosidase that removes the thio-linked glucose molecules from the glucosinolate. This phyto-compound possesses a wide range of biological activities such as anti-inflammatory [8], antioxidant [9], anticancer [10] and protects against neurodegenerative disorders [11]. In particular, in a primary culture of hippocampal neurons, treatment with MOR significantly promotes the early stages of neuronal differentiation increasing the number and length of dendrites and axon length and inducing synapse development [12]. Our group has already shown that MOR is able to improve the differentiation of periodontal ligament stem cells to neuronal cells [13]. MOR treatment accelerates the differentiation process in a short time (48 h) and the neuronal lineage is mainly induced. In another study, the periodontal ligament stem cells pre-treated with MOR promote beneficial effects in the inflammation response reducing the mitophagy process and the level of oxidative stress [14].

CBD is one of the non-psychoactive cannabinoids extracted from *Cannabis sativa*. It is a compound with a broad spectrum of potential therapeutic properties, including neuroprotective effects that are exerted by antioxidants [15] and anti-inflammatory [16] activities. In previous transcriptomic studies, our group showed that hGMSCs pre-treated with CBD prevented the activation of the NALP3-inflammasome, a cytosolic multiprotein oligomer, reducing the level of NALP3, CASP1 and IL-18 [17]. Moreover, CBD downregulates the pro-inflammatory and pro-apoptotic proteins. Our research group has previously demonstrated that the CBD pre-treatment shows in hGMSCs a downregulation of the genes involved in the phosphorylation of tau protein and in Aβ generation attenuating consequently the formation of neurofibrillary tangles and Aβ plaques that characterize AD [18]. In addition, the treatment with CBD downregulates the genes that are involved in the development of ALS and in its relevant cellular events such as oxidative stress, mitochondrial dysfunction and excitotoxicity [19]. Interestingly, in another study, we noticed that several genes involved in hGMSC cell proliferation, multipotency and renewal are regulated by CBD. Particularly, hGMSCs pretreated with CBD significantly activate several genes associated with neurogenesis [20].

In the present study, using Next-Generation Sequencing (NGS), we compared the transcriptomic profile of hGMSCs pre-treated with MOR (0.5 µM) or with CBD (5 µM) in order to evaluate analogies and differences in pro- and anti-inflammatory pathways. About the pro-inflammatory, we analyzed the transcriptomic profile of the genes involved in Tumor Necrosis Factor alpha (TNF-α), Interleukin-1 (IL-1) and Interleukin-6 (IL-6) pathways. Concerning anti-inflammation, we studied the TGF-β pathway.

Specifically, we want to evaluate if the treatment with MOR or CBD positively modulates the same inflammatory pathways in hGMSCs and, therefore, can effectively support the treatment of neurodegenerative diseases.

## 2. Results

### 2.1. Cell Characterization

**Figure 1:**
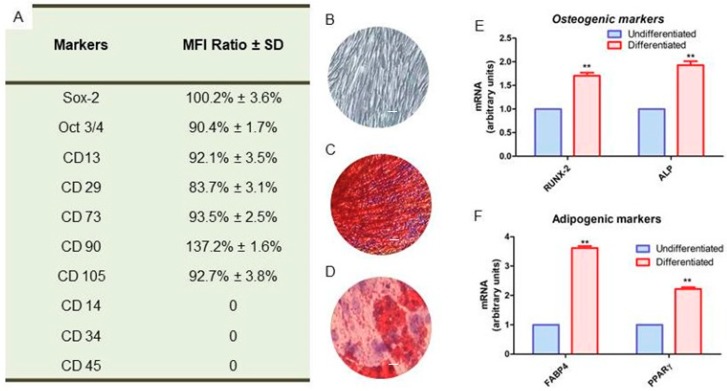
(**A**) Cytofluorimetric detection of positive and negative markers expressed in hGMSCs. (**B**) Confluent hGMSCs cultured on tissue culture dishes with a fibroblast lie morphology. (**C**) Alizarin Red S staining after 21 days of induction with osteogenic medium. (**D**) Oil Red O staining after 28 days of induction with adipogenic medium. (**E**) RT-PCR of osteogenic markers, as RUNX2 and ALP. (**F**) RT-PCR of adipogenic markers, as FABP4 and PPARγ. Scale bar: 10 µm. ** *p* <0.01.

According to the Dominici’s criteria to define human Mesenchymal Stem Cells [21], the hGMSCs were characterized by cytofluorimetric analysis. They showed the positivity for CD 13, 29, 73, 90, 105, Sox-2 and Oct3/4 while they were negative for CD 14, 34 and 45 (Figure 1A). When cells were plated into the tissue culture dishes, they showed a fibroblast-like morphology and the plastic adherent capacity (Figure 1B). To evaluate the ability to differentiate into osteogenic and adipogenic lineages, cells were stained with Alizarin Red S solution and with Oil Red O solution at the end of the induction period. Osteogenic differentiated hGMSCs showed the red positive calcium deposits (Figure 1C), while cells differentiated into adipogenic commitment showed red lipid droplets localized at the cytoplasmic level (Figure 1D). To confirm the differentiation process, reverse transcription polymerase chain reaction (RT-PCR) were performed. The genes that encode for RUNX-2, ALP, FABP4 and PPARγ were upregulated in differentiated cells when compared to the undifferentiated (Figure 1E,F).

### 2.2. Effects on Morphology and Viability of MOR and CBD Treatments

**Figure 2:**
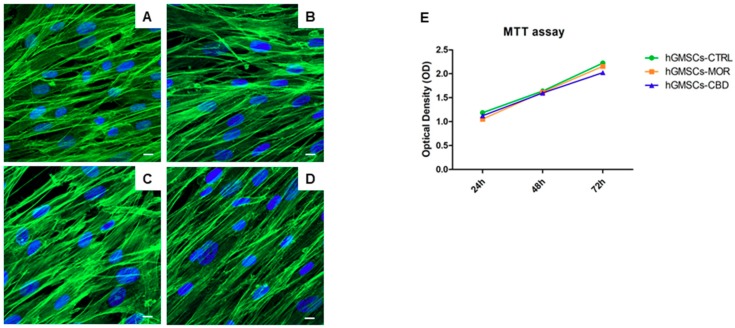
The immunofluorescence analysis for actin expression showed no morphological changes in hGMSCs-MOR (**B**) and in hGMSCs-CBD (**D**) when compared to hGMSCs-CTRL (**A**,**C**). Green fluorescence: cytoskeleton actin. Blue fluorescence: cell nuclei. Mag: 63X. Scale bar: 5 µm. The hGMSCs treated with MOR or CBD showed a similar proliferation rate of the untreated cells as demonstrated by MTT assay at 24, 48 and 72 h of culture (**E**).

The hGMSCs were treated with MOR at 0.5 µM and with CBD at 5 µM for 48 h. The in vitro biological features were evaluated using Confocal Laser Scanning Microscopy (CLSM). Untreated and treated hGMSCs were cultured in tissue culture dishes and they exhibited a similar fibroblast-like morphology and plastic-adherence to the substrate. MOR and CBD treatment did not lead to a cell morphological modification under CLSM observation (Figure 2B,D) when compared to the untreated cells (hGMSCs-CTRL, Figure 2A,C). The hGMSCs treated with MOR and CBD showed a similar proliferation rate of the untreated cells as demonstrated by 3-(4,5-dimethylthiazolyl-2)-2,5-diphenyltetrazoliumbromide (MTT) assays at 24, 48 and 72 h of culture (Figure 2E).

### 2.3. Transcriptomic Analysis

The differently expressed genes in hGMSCs-MOR or hGMSCs-CBD against the hGMSCs-CTRL were analyzed by means of Reactome database. In particular, we focused on the pathways triggered by the pro-inflammatory TNF-α, IL-1, IL-6 (Table 1) and the anti-inflammatory TGF-β (Table 2).

Table 1 counts 15 genes involved in TNF-α signaling among which *MAP3K7*, *CLIP3* and *CASP8* are downregulated in both the treatments. *TNFRSF1A*, *SPPL2A*, *RIPK1*, *USP21*, *CYLD*, *UBB* are downregulated in hGMSCs-MOR and upregulated in hGMSCs-CBD while *SHARPIN*, *CHUK*, *GNB2L1*, *RPS27A*, *UBA52* and *UBC* are deregulated in reverse. 

In the analysis, six genes that belong to IL-1 pathway are expressed. *MYD88*, *MAP3K7* and *SQSTM1* are downregulated while *MAP3K3* is upregulated in both the treatments. *IL1R1*, *CHUK* are upregulated in hGMSCs-MOR and downregulated in hGMSCs-CBD.

Moreover, the table counts 8 genes of the IL-6 signaling among which *IL6ST*, *STAT3* and *PIK3CD* are downregulated while *AKT1* is upregulated in both the treatments. *PIK3CA*, *PIK3CB* are downregulated in hGMSCs-MOR and upregulated in hGMSCs-CBD while *TYK2* is oppositely deregulated. *MTOR* is upregulated in hGMSCs-MOR but there is no statistical relevant deregulation in hGMSCs-CBD.

In our transcriptome, the anti-inflammatory TGF-β pathway counts of 14 genes. *FURIN*, *TGFBR1*, *SMURF2*, *STRAP* and *XPO1* are upregulated while *SMAD3* and *WWTR1* are downregulated in both the treatments. *NCOR1*, *PPP1CB*, *PPP1R15A* are downregulated in hGMSCs-MOR and upregulated in hGMSCs-CBD while *TGFBR2*, *FKBP1A*, *SMAD4*, *PPP1CA* show opposite behavior.

**Figure 3:**
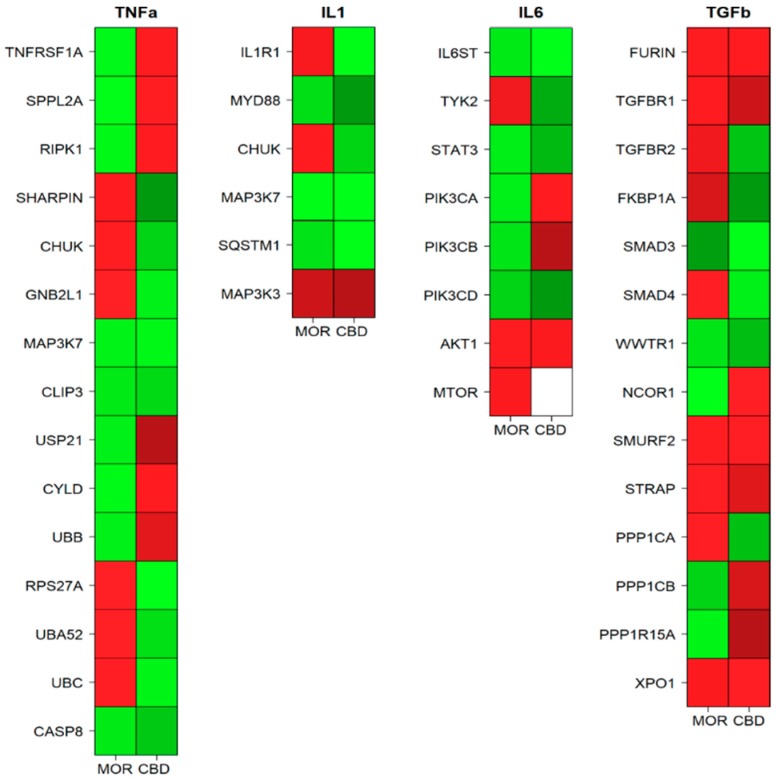
Heatmaps of the genes involved in the inflammation pathways among the hGMSCs-MOR (MOR) or hGMSCs-CBD (CBD) treatments sorted by their intervention in the pathway. The green color represents a downregulation while the red color an upregulation. The white color stands for no statistically significant change.

In addition, Figure 3 represents a heatmap for the aforementioned pathways in which all the genes represented in Table 1 and in Table 2 are included. The heatmap shows how TNF-α signaling is downregulated asynchronously in the treatments. Moreover, hGMSCs-CBD downregulate most of the genes involved in the IL-1 pathway while hGMSCs-MOR is not able to downregulate its receptor. Furthermore, the IL-6 signaling is totally downregulated by CBD in the pro-inflammatory JAK/STAT pathway while MOR enhances the pro-survival effects of PI3K/AKT/mTOR. Finally, most of the genes in the TGF-β pathway are upregulated in both hGMSCs-MOR and hGMSCs-CBD.

### 2.4. Protein Expression

**Figure 4:**
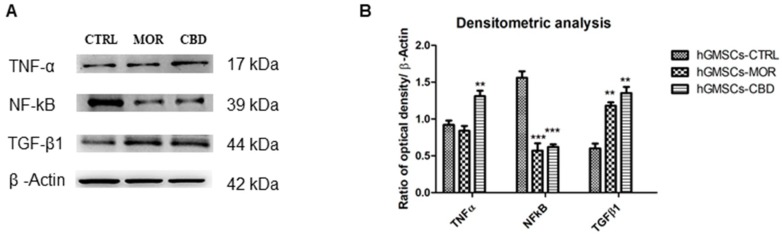
(**A**) Protein expression evaluated by means Western blot analysis of TNF-α, NF-ĸB, TGF-β1 and β-Actin. (**B**) Bars graph of densitometric analysis. *** *p* < 0.001; ** *p* < 0.01.

Western blotting analysis was performed in order to confirm the transcriptomic data. In Figure 4, protein-specific bands were reported. In particular, NF-ĸB reduction was shown in hGMSC-MOR and in hGMSCs-CBD when compared to the in hGMSCs-CTRL. The TNF-α showed a decreased level in hGMSCs-MOR while it was overexpressed in hGMSCs-CBD. At the same time, TGF-β1 showed an increased expression in hGMSCs-MOR and in hGMSCs-CBD (Figure 4A,B).

## 3. Discussion

Inflammatory responses play a central role in the ethiopathogenesis of many neurodegenerative diseases and stem cell-based therapies could provide an alternative approach to support the regeneration of the damaged tissues [22]. Numerous studies have shown that the hGMSCs regulate the immune system by the inhibition of the proliferation of T and B cells [23], reducing apoptosis and influencing the production of pro-inflammatory and anti-inflammatory cytokines [24]. 

In this study, hGMSCs were pre-treated with MOR (0.5 µM) or CBD (5 µM), two phytocompounds extensively studied for their antioxidant [25] and anti-inflammatory [17,26] properties. Specifically, the dose of CBD for the treatment of the hGMSCs was chosen based on previous studies in which we have already demonstrated that it is the lowest dose that effectively exerts anti-inflammatory action [20,25]. In contrast, hGMSCs-MOR were treated with a low concentration of MOR because our group showed that higher doses do not change the transcriptomic profile of the hGMSCs [13]. The MOR or CBD treatment of hGMSCs does not induce cytotoxicity as highlighted in MTT (Figure 2E) and does not modify the morphology of the cells (Figure 2B,D). Here, we focused on the pathways mediated by TNF-α, IL-1, IL-6 and TGF-β.

TNF-α is a pro-inflammatory cytokine that interacts with TNF receptor 1 (TNFR1), expressed in most tissues [27]. In our transcriptome, the *TNFRSF1A* gene, which encodes for TNFR1, is downregulated in hGMSCs-MOR and upregulated in hGMSCs-CBD. The result matches the Western blot analysis (Figure 4) that shows a reduced level of TNF-α in hGMSCs-MOR but not in hGMSCs-CBD suggesting a drop in TNF-α signaling after MOR treatment. When TNF-α binds TNFR1, it induces the activation of different biological pathways mediated by complexes I and II [28]. Both the complexes are activated from the Receptor-Interacting Protein Kinase 1 (RIPK1) protein, that along with TNFR1, plays the main role in the biological response [29]. RIPK1 is encoded by the *RIPK1* gene that is downregulated in hGMSCs-MOR and upregulated in hGMSCs-CBD. In hGMSCs-MOR, the simultaneous downregulation of *TNFRSF1A* and *RIPK1* hinders complexes I and II activation and strengthens the hypothesis of a reduction in the intracellular response.

In particular, complex I actives inflammation, tissue degeneration, cell proliferation or survival. As a result of the inflammatory process, RIPK1 seems to promote the pro-inflammatory response by NF-ĸB or the cascade activation of kinases [30]. Specifically, RIPK1 needs LUBAC complex to exert its activity, which adds ubiquitin chains to RIPK1. SHARPIN encoded by the *SHARPIN* gene is an additional component of LUBAC that linearly ubiquitinates IKK-γ, the regulatory subunit of the Inhibitor of kB Kinase (IKK) complex [31]. In this way, SHARPIN and RIPK1-ubiquitinated activate the IKK complex leading to the phosphorylation of the IκBα proteins. This phosphorylation triggers the degradation of IκBα. Consequently, the NF-κB is liberated from IκBα and translocates into the nucleus where it exerts its action [32]. Therefore, the propagation of the inflammatory signal needs the functional activation of both *SHARPIN* and *RIPK1* that are alternatively downregulated in hGMSCs-MOR and hGMSCs-CBD. According to this result, the Western blot analysis shows reduced levels of NF-κB in both the treatments (Figure 4). *CHUK* gene, expressed in our transcriptome, is downregulated in hGMSCs-CBD and upregulated in hGMSCs-MOR. CHUK, that encodes for IKKα, is another component of the IKK complex [33] likely involved in cell differentiation [34]. In the inflammatory response, the upregulation of *CHUK* in hGMSCs-MOR is interesting since it acts as part of the canonical IKK complex in the conventional pathway of NF-κB activation [35]. The complex IKK is also modulated by RACK1 protein, encoded by *GNB2L1* gene that in our transcriptomes is upregulated in hGMSCs-MOR and downregulated in hGMSCs-CBD. RACK1 is a scaffold protein that can regulate cell growth and mediate stress signals. In particular, *GNB2L1* gene is deregulated similarly to CHUK in both the treatments suggesting the inhibition of NF-ĸB because of the direct interaction of RACK1 with IKK [36].

Moreover, CLIP3 is an adaptor protein involved in recruiting CYLD into the TNFR1 signaling that facilitates CYLD-mediated deubiquitination of RIPK1 in TNF-α signaling [37]. Our results also show that *CLIP3*, that encodes for CLIP3 proteins, is downregulated in both the treatments. Instead, *USP21* and *CYLD* are downregulated in hGMSCs-MOR and upregulated in hGMSCs-CBD. *USP21* encodes for the ubiquitin-specific peptidase 21 while *CYLD* encodes for CYLD lysine 63 deubiquitinase. They modulate negatively the TNF-α signaling removing the ubiquitins from the RIPK1, as previously described [38]. Noteworthy, the upregulation of both *USP21* and *CYLD* in hGMSCs-CBD should lead to a lower amplification of the signal mediated by the TNF-α. Moreover, in our transcriptome, the genes encoding for the ubiquitins are *UBB*, *RPS27A*, *UBA52* and *UBC*. *UBB* is downregulated in hGMSCs-MOR and upregulated in hGMSCs-CBD while the other ubiquitins are deregulated in the opposite manner. The ubiquitins act as a scaffold for the activation of the complex I and their overall downregulation mediated by hGMSCs-CBD could be useful to reduce the chronic inflammation stimuli [39]. This is another mechanism that shows how CBD exerts its anti-inflammatory action negatively regulating the TNF- α pathway.

RIPK1-ubiquinated can also promote the activation of map kinases by phosphorylation of TAK1 protein, encoded by *MAP3K7* gene that is downregulated in hGMSCs-MOR and hGMSCs-CBD. TAK1 is a precursor protein of the Mitogen Activated Protein Kinase (MAPK) pathway and intriguing the downregulation of *MAP3K7* reduces the activation of the cascade that leads to inflammation [40]. 

RIPK1 is also implied in the apoptosis without recruiting NF-kB when it is associated in the complex II. It recruits the caspase 8 and, finally, promote the cell death [41]. The caspase 8 is an initiator of the apoptosis but it plays also an essential role in the inflammation [42]. The downregulation of *CASP8* in both the treatments and the MTT assay (Figure 2E) suggest the reduction of the cell death in hGMSCs-MOR and hGMSCs-CBD.

In parallel, a new scenario hypothesizes the activation of NF-ĸB by the Signal Peptidase Like 2A (SPPL2A) protein. Indeed, SPPL2A is an intramembrane protease that seems to promote the inflammatory response in activated dendritic cells by cleaving the TNF-α [43]. It is encoded by *SPPL2A* gene and it is downregulated in hGMSCs-MOR and upregulated in hGMSCs-CBD. The lower expression in hGMSCs-MOR suggests the reduction of the inflammation mediated by NF-ĸB.

IL-1 is a family of cytokines including IL-1α and IL-1β that are active in inflammatory processes [44]. IL-1 triggers its effects after the interaction with the membrane receptor Interleukin 1 Receptor type 1 (IL1R1) encoded by *IL1R1* gene that is upregulated in hGMSCs-MOR and downregulated in hGMSCs-CBD. The interaction between IL-1 and IL1R1 causes a conformational change of the receptor that determines its association with the adaptive protein MyD88. MyD88, encoded by the *MYD88* gene, is a signal transducer that promotes the subsequent NF-κB-mediated transcription [45]. Our results show that both hGMSCs-MOR and hGMSCs-CBD downregulate the expression of the gene *MYD88* with consequent reduction in signal transduction. Similarly to TNF-α signaling, the IL-1 pathway modulates *MAP3K7* (TAK1) and *CHUCK* (IKKα) genes. Therefore, the downregulation of *MAP3K7* in both our experimental groups matches the downregulation of *MYD88* and it suggests the reduction NF-ĸB activation. In addition, our transcriptomic profiles show that *SQSTM1* gene is downregulated both in hGMSCs-MOR and hGMSCs-CBD whereas *MAP3K3* gene is upregulated in both the treatments. *SQSTM1* encodes for p62, a multifunctional protein that acts as an adaptive scaffold/protein associated with Mitogen-Activated Protein Kinase Kinase Kinase 3 (MAP3K3) [46]. MAP3K3 is encoded by the *MAP3K3* gene and it is a component of a protein kinase cascade that mediates activation of NF-ĸB [47]. Therefore, the downregulation of the *SQSTM1* gene in both experimental groups leads to less formation of the MAP3K3/p62 complex and, consequently, it contributes to reduce NF-ĸB effects.

IL-6 is a pro-inflammatory cytokine that contributes to host defense by means of the stimulation of pro-inflammatory responses and immune reactions [48]. When the IL-6 receptor (IL6R) is in complex with the Interleukin 6 Signal Transducer (IL6ST) protein, it can mediate the signal transduction. IL6ST is encoded by *IL6ST* that in our results is downregulated both in hGMSCs-MOR and hGMSCs-CBD. IL6ST selectively engages a member of the Janus Kinase family (JAK) [49] like TYK2. *TYK2* gene, that encodes for TYK2, is upregulated in hGMSCs-MOR but downregulated in hGMSCs-CBD. TYK2 is a tyrosine kinase that phosphorylates IL6ST creating a binding site for the transcription factor STAT3. STAT3 is encoded by the *STAT3* gene and it is downregulated in both the treatment. As soon as STAT3 binds IL6ST, it is phosphorylated by TYK2 [50]. The phosphorylation of STAT3 promotes its dimerization and translocation into the nucleus where it starts transcription. The simultaneous downregulation of *IL6ST*, *TYK2* and *STAT3* strongly suggests the inhibition of the JAK/STAT pathway in hGMSCs-CBD. In parallel, IL-6 can also trigger the activation of the non-classic PI3K/AKT/mTOR pathway. Specifically, the MOR pretreatment of hGMSCs downregulates *PIK3CA*, *PIK3CB,* and *PIK3CD* that encode for the catalytic subunits of PI3K, restricting the activation of this cascade pathway by IL-6. On the contrary, the CBD pretreatment downregulates only *PIK3CD* but upregulates *PIK3CB* and *PIK3CD*. Nevertheless, both the treatments upregulate *AKT1*. It encodes AKT Serine/Threonine Kinase 1 that promotes cell survival by inhibition of apoptosis [51]. Interestingly, *MTOR* gene, that encode mTOR, is upregulated in hGMSCs-MOR whereas in hGMSCs-CBD there is not any statistically significant change in its expression level. The role of mTOR is quite important because it is implied in cell proliferation and neurogenesis [52]. 

The hGMSCs-MOR and hGMSCs-CBD modulate the anti-inflammatory pathway mediated by TGF-β that is involved in proliferation, differentiation, migration, cellular homeostasis and neuronal survival [53]. The FURIN protein, encoded by the *FURIN* gene, promotes the functional activation of the TGF-β by the cleavage of its N-terminal [54]. In our results, *FURIN* is upregulated in both treatments. The activated TGF-β binds the TGF-β Receptor type I (TGFβRI) and type II (TGFβRII), encoded by *TGFBR1* and *TGFBR2* genes, respectively [55]. Our results show that *TGFBR1* is upregulated in both the treatment while *TGFBR2* is upregulated only in hGMSCs-MOR. Interestingly, the simultaneous upregulation of *FURIN*, *TGFBR1* and *TGFBR2* genes makes to hypothesize the complete trigger of the signaling in hGMSCs-MOR whereas the downregulation of *TGFBR2* suggests a partial trigger in hGMSCs-CBD. Moreover, the Western blot analysis in Figure 4 shows higher level of TGF-β1 in both treatments. FKBP1A is a cis–trans peptidyl-prolyl isomerase that prevents TGFβRI phosphorylation by TGFβRII [56]. It is encoded by the *FKBP1A* gene that is upregulated in hGMSCs-MOR and downregulated in hGMSCs-CBD. When TGF-β binds TGFβRII, TGFβRI complexes with TGFβRII that auto-phosphorylates itself and trans-phosphorylates TGFβRI. TGFβRI phosphorylates SMAD3 that acquires affinity towards SMAD4 protein that finally translocates into the nucleus in which it promotes transcription [57]. SMAD3 and SMAD4 (SMADs) are signal transducers proteins encoded, respectively, by *SMAD3* and *SMAD4*. *SMAD3* is downregulated in hGMSCs-MOR and in hGMSCs-CBD while *SMAD4* only in hGMSCs-CBD. Even if *SMAD3* is downregulated in both the treatments, the overexpression of *SMAD4* in hGMSCs-MOR suggests that the SMAD3 phosphorylation could occur. Inside the nucleus, the transcriptional activity of the SMADs is mediated by WWTR1 [58]. WWTR1 is encoded by the *WWTR1* gene and it is downregulated in hGMSCs-MOR and hGMSCs-CBD. The modulation of the TGF-β signal can be differently mediated by NCOR1, SMURF2, STRAP, PP1, XPO1 proteins. NCOR1, SMURF2 and STRAP proteins are, respectively, encoded by *NCOR1*, *SMURF2* and *STRAP* genes. NCOR1 is a nuclear receptor corepressor that stops the transcription activity of SMADs [59]. SMURF2 is an E3 ubiquitin-protein ligase that adds ubiquitin on SMADs, among which SMAD3, promoting their degradation [60]. STRAP is a Serine–threonine kinase receptor-associated protein that binds the receptors TGFβRI and TGFβRII hindering the propagation of the signal [61]. Both *SMURF2* and *STRAP* genes are upregulated in hGMSCs-MOR and hGMSCs-CBD while *NCOR1* only in hGMSCs-CBD. The signal induced by TGF-β can be also negatively modulated by Protein Phosphatase 1 (PP1) that removes the phosphate groups from TGFβR1 [62]. In particular, our transcriptomes express two catalytic subunits encoded by *PPP1CA* and *PPP1CB* genes and a regulatory subunit encoded by *PPP1R15A* gene. *PPP1CA* is upregulated in hGMSCs-MOR and downregulated in hGMSCs-CBD whereas *PPP1R15A* and *PPP1CB* are modulated in reverse. The conclusion of the signal is also mediated by the exportin XPO1 that promotes the translocation of the transcripts from the nucleus to the cytosol. XPO1 is encoded by *XPO1* gene that is upregulated in both the treatments. Interestingly, the upregulation of *XPO1*, *STRAP* and *SMURF2* genes matches with the upregulation of *FURIN* and *TGFBR1* genes both in hGMSCs-MOR and hGMSCs-CBD. It suggests the conclusion of the signal propagation by the treated cells in order to avoid prolonged stimuli.

## 4. Materials and Methods

### 4.1. Ethics Statement

The study followed the guidelines of the Helsinki Declaration (2013). The stem cells were collected under the approval of the Ethical Committee (number 266/17.04.2014) at the Medical School, “G. d’Annunzio” University, Chieti, Italy. The three recruited healthy individuals signed the informative consent form before tissue collection.

### 4.2. Compounds Isolation and Purification

The MOR was extracted from the seeds of *Moringa oleifera* at the CREA-AA (Bologna Laboratory), as previously described by Brunelli et al. [63]. MOR was purified by anion exchange and size exclusion chromatography. The MOR was purified by High-performance liquid chromatography (HPLC) obtaining a purity of about 99% (based on peak area value) [64]. CBD was extracted by flowerheads of *Cannabis sativa L.* and it was obtained from a greenhouse cultivation at the Council for Research and Experimentation in Agricolture-Research Center for Industrial Crops (CREA-CIN) in Rovigo, using established methods previously described by Taglialatela et al. [65]. The isolation and purification of cannabinoid was done in accordance with their legal status (Authorization SP/106 23/05/2013 of the Ministry of Health, Rome, Italy). The extracted CBD had a purity >99% and it did not show any trace of THC.

### 4.3. Cell Isolation

The hGMSCs have been obtained from the gingival tissue collected during a routine oral surgery procedure as previously described [66]. Healthy patients scheduled to remove the third molar for orthodontic purposes were enrolled in the study. In order to exclude most keratinocytes resident in the gingival, the gingival specimens were completely de-epithelialized with a scalpel. The connective tissues were grinded and after washed several times with phosphate buffered saline (PBS; EuroClone, Milan, Italy). Tissue explants were cultured in tissue culture dishes with Mesenchymal Stem Cells Growth Medium-Chemically Defined (Lonza, Basel, Switzerland). The medium was replaced with a fresh one every two days. After two weeks of culture, cells spontaneously migrated from tissue explants. All experiments were performed with cells at 2nd passage. After the isolation process, cells were treated with MOR (0.5 µM) or CBD (5 µM) for 48h.

### 4.4. Cell Characterization

To evaluate the mesenchymal features of hGMSCs, the cytofluorimetric detection and mesengenic differentiation were performed. The cytofluorimetric analysis was assayed as previously described [67]. Expression of Sox-2, Oct3/4, CD13, CD14, CD29, CD34, CD45, CD73, CD90, and CD105 was evaluated on hGMSCs. The analysis was performed by using FACStarPLUS flow cytometry system and the FlowJo™ software (TreeStar, Ashland, OR, USA). To assess the ability to differentiate into osteogenic and adipogenic commitment, hGMSCs were maintained under osteogenic and adipogenic conditions for 21 and 28 days, respectively, as reported by Pizzicannella, et al. [68]. To evaluate the formation of mineralized precipitates and lipid vacuoles, after the differentiation period, alizarin red and Oil red O staining were performed on undifferentiated and differentiated cells. Inverted light microscopy Leica DMIL (Leica Microsystem, Milan, Italy) was used for samples observations. To validate the ability to differentiate into osteogenic and adipogenic lineages, the expression of RUNX-2, ALP, FABP4, and PPARγ were evaluated by RT-PCR as reported by Pizzicannella, et al. [68]. Commercially available TaqMan Gene Expression Assays (RUNX-2 Hs00231692_m1; ALP Hs01029144_m1; FABP4 Hs01086177_m1; PPARγ Hs01115513_m1) and the Taq-Man Universal PCR Master Mix (Applied Biosystems, Foster City, CA, USA) were used according to standard protocols. Beta-2 microglobulin (B2M Hs99999907_m1) (Applied Biosystems) was used for template normalization. Real-time PCR was performed in three independent experiments, and duplicate determinations were carried out for each sample.

### 4.5. Cell Culture and Treatments

The hGMSCs were cultured in monolayer using DMEM-High Glucose (SIGMA–ALDRICH, Co.) containing 10% fetal bovine serum (FBS) (Sigma–Aldrich Co. Ltd). The cells were grown in a logarithmic phase at 37 °C in a moisturized atmosphere of 5% CO2 and 95% air. The hGMSCs grown until at 70–80% confluence were incubated for 48h in the medium containing 5 µM CBD (dissolved in 0.1% DMSO) or 0, 5 µM of MOR (dissolved in 0.1% DMSO). The hGMSCs treated with 0.1% DMSO were included as controls. After 48 h incubation, cells were harvested for the other analysis. 

The experiment was repeated in triplicates.

### 4.6. Cell Viability Assay

The proliferation ability of hGMSCs cultured with or without MOR (0.5 μM) or with CBD (5  μM) for 48 h, were determined using the 3-(4,5-dimethylthiazolyl-2)-2,5-diphenyltetrazoliumbromide (MTT) method. At the end of the treatment, cells were incubated with medium containing MTT (0.5 mg/mL; Sigma-Aldrich) at 37 °C for 4 h. Living cells are able to reduce yellow tetrazole to purple insoluble formazan. The formed formazan crystals were dissolved in acid isopropanol at 37 °C for 1 h. The absorbance level of MTT cleavage was directly proportional to the number of viable cells and indirectly indicate proliferation rate after MOR or CBD treatment. Supernatants were read at 650 nm wavelength using a microplate reader (Synergy HT, BIoTek Instruments, VT).

### 4.7. Morphological Analyses

To evaluate the morphological features in untreated and treated cells, samples were processed for immunofluorescence detections. Untreated hGMSCs and treated hGMSCs with MOR and CBD, were cultured on eight-well chamber slides and then were fixed using 4% paraformaldehyde diluted in 0.1 M sodium phosphate buffer (PBS, Lonza). After the fixation step, cells were permeabilized with 0.5% Triton X-100 in PBS for 10 min, followed by blocking with 5% non-fat dried milk in PBS for 30 min [69]. Cells were incubated by Alexa Fluor 488 phalloidin green fluorescence conjugate (1:400, Molecular Probes, Invitrogen, Eugene, OR, USA) in order to mark the cytoskeleton actin. After immunofluorescence labelling cells were washed and incubated with TOPRO (1:200, Molecular Probes) for 1 h at 37 °C for nuclei evaluation [70]. Samples were observed under a Zeiss LSM800 confocal system (Zeiss, Jena, Germany). 

### 4.8. RNA Extraction and NGS Analysis

The extraction of the total RNA was performed with the Reliaprep RNA cell Miniprep System (Promega, Madison, WI, USA). The total RNA was fragmented according to TruSeq RNA Access library kit protocol (Illumina, San Diego, CA, USA). The SuperScript II Reverse transcriptase (Invitrogen, Carlsbad, CA, USA) was used to synthesize the cDNA. In order to facilitate adaptor ligation in the next step, the 3’end of the cDNA were adenylated and then ligation of indexing adaptors was performed. The PCR was used to amplify the library and the AMPure XP beads (Beckman Coulter, Brea, CA) for the clean-up. PCR (15 cycles of 98 °C for 10 s, 60 °C for 30 s and 72 °C for 30 s) was performed to select those DNA fragments that have adapter molecules on both ends and to amplify the amount of DNA in the library. After validation of the library, the first hybridization step (18 cycles of 1 min incubation, starting at 94 °C, and then decreasing 2 °C per cycle) was performed using exome capture probes. Then, to capture probes hybridized to the target regions, streptavidin coated magnetic beads were used; then, the enriched libraries were eluted and prepared for a second round of hybridization. The second hybridization was required to ensure high specificity of the capture regions. A second capture with streptavidin-coated beads was performed, the enriched libraries were eluted and cleaned up by AMPure XP beads. The second PCR amplification step was performed by ten cycles. The library was quantified by qPCR KAPA Library Quantification Kit-Illumina/ABI Prism^®^ (Kapa Biosystems, Inc., Wilmington, MA, USA). Agilent High Sensitivity DNA Kit on a Bioanalyzer was used for validation of libraries. The size range of the DNA fragments was measured to be in the range of 200–650 bp and peaked around 250 bp. The library was finally normalized at 12 pM and sequenced using MiSeq Reagent Kit v3 by Illumina MiSeq Instrument in single read mode. The transcriptome profile was aligned against the reference genome “UCSC hg19” and the fold change of the differentially expressed genes of each treatment against hGMSCs-CTRL were evaluated by Cufflinks package. The experiment was repeated in triplicate.

### 4.9. Bioinformatics Analysis and Database Inspection

The biological role of differently expressed gene were inspected taking advantage of the Reactome database from the online website (https://reactome.org/). In particular, we focused on the pathways “TNF signaling”, “Interleukin-1 signaling”, “Interleukin-6 signaling” and “Signaling by TGF-beta Receptor Complex” in order to study TNF-α, IL-1, IL-6 and TGF-β pathways. 

### 4.10. Western Blot Analysis

The proteins (30 μg) obtained from all the samples were processed as previously described [71]. Proteins were separated on SDS-PAGE and subsequently transferred to nitrocellulose sheets using a semidry blotting apparatus. The sheets were saturated for 60 min at 37 °C in blocking buffer (1xTBS, 5% milk, 0.05% Tween-20), then incubated overnight at 4 °C in blocking buffer containing primary antibodies to NF-ĸB (1:1000, Cell Signaling Technology, Danvers, MA, USA), TGF-β1 (1:750, Abcam, Cambridge, UK), TNF-α (1:1000, Cell Signaling Technology) and β-actin (1:1000, Santa Cruz Biotechnology, Santa Cruz, CA, USA). After four washes in TBS containing 0.1% Tween-20, samples were incubated for 30 min at room temperature with peroxidase-conjugated secondary antibody diluted 1:1000 in 1× TBS, 5% milk, 0.05% Tween-20. Bands were visualized by the ECL method. The level of recovered protein was measured using the Bio-Rad (Bio-Rad Laboratories, Hercules, CA, USA) Protein Assay according to the manufacturer’s instructions.

## 5. Conclusions

The pretreatment of hGMSCs with CBD or MOR has an anti-inflammatory activity enhancing the TGF-β pathway, involved in the regulation of multiple biological processes, including inflammation. Concerning the pro-inflammatory pathways, TNF-α signaling is reduced from the beginning in hGMSCs pretreated with MOR as confirmed by Western blot analysis, while CBD exerts its activity after the process starts.

Contrarily, the treatment with CBD selectively inhibits the IL-1 pathway while MOR is less efficient. Furthermore, IL-6 signaling is modulated by CBD that downregulates the pro-inflammatory JAK/STAT and by MOR that enhances the PI3K/AKT/mTOR pathway, which leads to cell survival and proliferation.

## Figures and Tables

**Table 1:** Genes of pro-inflammatory pathways in hGMSCs-MOR and hGMSCs-CBD.

Gene	Name	Fold Change hGMSCs-MOR	Fold Change hGMSCs-CBD	Q-Value hGMSCs-MOR	Q-Value hGMSCs-CBD
*AKT1*	AKT serine/threonine kinase 1	0.23	0.34	2.50 × 10^−3^	9.79 × 10^−5^
*CASP8*	Caspase 8	−0.65	−1.41	2.11 × 10^−4^	9.79 × 10^−5^
*CHUK*	Component of inhibitor of nuclear factor kappa B kinase complex	0.35	−1.19	9.77 × 10^−3^	9.79 × 10^−5^
*CLIP3*	CAP-Gly domain containing linker protein 3	−0.71	−1.03	2.11 × 10^−4^	9.79 × 10^−5^
*CYLD*	CYLD lysine 63 deubiquitinase	−0.32	0.47	2.11 × 10^−4^	9.79 × 10^−5^
*GNB2L1*	Receptor for activated C kinase 1	0.13	−0.51	1.43 × 10^−3^	9.79 × 10^−5^
*IL1R1*	Interleukin 1 receptor type 1	0.43	−0.46	2.11 × 10^−4^	1.91 × 10^−3^
*IL6ST*	Interleukin 6 signal transducer	−0.43	−0.12	2.11 × 10^−4^	2.18 × 10^−2^
*MAP3K3*	Mitogen-activated protein kinase kinase kinase 3	0.74	0.87	2.11 × 10^−4^	9.79 × 10^−5^
*MAP3K7*	Mitogen-activated protein kinase kinase kinase 7	−0.47	−0.38	2.11 × 10^−4^	1.27 × 10^−3^
*MTOR*	Mechanistic target of rapamycin kinase	0.34	0.0	2.11 × 10^−4^	>5 × 10^−2^
*MYD88*	MYD88 innate immune signal transduction adaptor	−1.05	−2.26	2.11 × 10^−4^	9.79 × 10^−5^
*PIK3CA*	phosphatidylinositol-4,5-bisphosphate 3-kinase catalytic subunit alpha	−0.37	0.32	1.74 × 10^−3^	1.35 × 10^−3^
*PIK3CB*	phosphatidylinositol-4,5-bisphosphate 3-kinase catalytic subunit beta	−0.52	0.75	1.35 × 10^−2^	9.79 × 10^−5^
*PIK3CD*	phosphatidylinositol-4,5-bisphosphate 3-kinase catalytic subunit delta	−0.79	−1.59	2.11 × 10^−4^	9.79 × 10^−5^
*RIPK1*	Receptor interacting serine/threonine kinase 1	−0.37	0.50	2.44 × 10^−2^	2.29 × 10^−3^
*RPS27A*	Ribosomal protein S27a	0.16	−0.16	2.11 × 10^−4^	9.79 × 10^−5^
*SHARPIN*	SHANK associated RH domain interactor	0.50	−2.45	2.70 × 10^−2^	1.90 × 10^−4^
*SPPL2A*	Signal peptide peptidase like 2A	−0.22	0.32	2.56 × 10^−2^	3.64 × 10^−4^
*SQSTM1*	Sequestosome 1	−0.94	−0.44	2.11 × 10^−4^	9.79 × 10^−5^
*STAT3*	Signal transducer and activator of transcription 3	−0.40	−1.15	2.11 × 10^−4^	9.79 × 10^−5^
*TNFRSF1A*	TNF receptor superfamily member 1A	−0.35	0.38	2.11 × 10^−4^	9.79 × 10^−5^
*TYK2*	Tyrosine kinase 2	0.41	−1.33	7.66 × 10^−4^	9.79 × 10^−5^
*UBA52*	Ubiquitin A-52 residue ribosomal protein fusion product 1	0.14	−0.92	2.11 × 10^−4^	9.79 × 10^−5^
*UBB*	Ubiquitin B	−0.52	0.70	2.11 × 10^−4^	9.79 × 10^−5^
*UBC*	Ubiquitin C	0.30	−0.43	2.11 × 10^−4^	9.79 × 10^−5^
*USP21*	Ubiquitin specific peptidase 21	−0.51	0.98	3.46 × 10^−2^	2.77 × 10^−4^

The genes represented in the column Gene are associated to the name retrieved in HUGO Gene Nomenclature Committee website (column Name). Fold Change hGMSCs-MOR shows the fold change obtained by Log_2_(hGMSCs-MOR/hGMSCs-CTRL) while Fold Change hGMSCs-CBD highlights the fold change obtained by Log_2_(hGMSCs-CBD/hGMSCs-CTRL). The Q-value hGMSCs-MOR and Q-value hGMSCs-CBD were used to choose the level of significance (<0.05). All the fold change values are rounded to the second decimal digit while the Q-values are approximated with scientific notation.

**Table 2:** Genes of anti-inflammatory pathway in hGMSCs-MOR and hGMSCs-CBD.

Gene	Name	Fold Change hGMSCs-MOR	Fold Change hGMSCs-CBD	Q-Value hGMSCs-MOR	Q-Value hGMSCs-CBD
*FKBP1A*	FKBP prolyl isomerase 1A	0.77	−0.78	2.11 × 10^−4^	9.79 × 10^−5^
*FURIN*	Furin, paired basic amino acid cleaving enzyme	0.37	0.39	1.27 × 10^−3^	9.79 × 10^−5^
*NCOR1*	Nuclear receptor corepressor 1	−0.17	0.13	8.18 × 10^−3^	1.71 × 10^−2^
*PPP1CA*	Protein phosphatase 1 catalytic subunit alpha	0.20	−0.55	2.41 × 10^−2^	9.79 × 10^−5^
*PPP1CB*	Protein phosphatase 1 catalytic subunit beta	−0.44	0.78	2.11 × 10^−4^	9.79 × 10^−5^
*PPP1R15A*	Protein phosphatase 1 regulatory subunit 15A	−0.24	1.01	3.32 × 10^−2^	9.79 × 10^−5^
*SMAD3*	SMAD family member 3	−0.75	−0.19	2.11 × 10^−4^	1.91 × 10^−3^
*SMAD4*	SMAD family member 4	0.18	−0.27	3.32 × 10^−2^	2.77 × 10^−4^
*SMURF2*	SMAD specific E3 ubiquitin protein ligase 2	0.23	0.19	1.40 × 10^−2^	9.79 × 10^−5^
*STRAP*	Serine/threonine kinase receptor associated protein	0.20	0.70	4.56 × 10^−2^	9.79 × 10^−5^
*TGFBR1*	Transforming growth factor beta receptor 1	0.44	0.56	7.66 × 10^−4^	9.79 × 10^−5^
*TGFBR2*	Transforming growth factor beta receptor 2	0.86	−0.52	2.11 × 10^−4^	9.79 × 10^−5^
*WWTR1*	WWTR1	−0.34	−0.57	2.94 × 10^−3^	9.79 × 10^−5^
*XPO1*	Exportin 1	0.46	0.21	2.11 × 10^−4^	1.59 × 10^−3^

The genes represented in the column Gene are associated to the name retrieved in HUGO Gene Nomenclature Committee website (column Name). Fold Change hGMSCs-MOR shows the fold change obtained by Log_2_(hGMSCs-MOR/hGMSCs-CTRL) while Fold Change hGMSCs-CBD highlights the fold change obtained by Log_2_(hGMSCs-CBD/hGMSCs-CTRL). The Q-value hGMSCs-MOR and Q-value hGMSCs-CBD were used to choose the level of significance (<0.05). All the fold change values are rounded to the second decimal digit while the Q-values are approximated with scientific notation.

## Abbreviations

AD	Alzheimer’s Disease
PD	Parkinson’s Disease
MS	Multiple Sclerosis
ALS	Amyotrophic Lateral Sclerosis
hGMSCs	human Gingival Mesenchymal Stem Cells
MOR	Moringin
CBD	Cannabidiol
IL-10	Interleukin 10
IL-1	Interleukin 1
IL-6	Interleukin-6
TNF-α	Tumor Necrosis Factor alpha
IL6R	IL-6 Receptor
TGF-β	Transform Growth Factor beta
hGMSCs-MOR	hGMSCs treated with MOR
hGMSCs-CBD	hGMSCs treated with CBD
RT-PCR	reverse transcription polymerase chain reaction
CLSM	Confocal Laser Scanning Microscopy
hGMSCs-CTRL	hGMSCs untreated
TNFR1	TNF receptor 1
RIPK1	Receptor Interacting Protein Kinase 1
IKK	IκB kinase
IL1R1	IL-1 receptor type I
TIR	Toll-IL-1-Receptor
MAPK	Mitogen-Activated Protein Kinase
MAP3K3	Mitogen-Activated Protein Kinase Kinase Kinase 3
JAKs	Janus kinases
TGFβR1	TGFβ Receptor type 1
TGFβR2	TGFβ Receptor type 2
PP1	Protein Phosphatase 1
PBS	Phosphate Buffered Saline
MTT	3-(4,5-dimethylthiazolyl-2)-2,5-diphenyltetrazoliumbromide
